# STRESS AND IDENTITY FOR LGBTQ+ HEALTH: MODELING RISK AND RESILIENCE

**DOI:** 10.1093/geroni/igae098.3726

**Published:** 2024-12-31

**Authors:** Erin Clancy, M Aaron Guest, Aanika Dhawan, Rachel Koffer

**Affiliations:** Arizona State University, Tempe, Arizona, United States; Arizona State University, Phoenix, Arizona, United States; Arizona State University, Tempe, Arizona, United States; Arizona State University, Phoenix, Arizona, United States

## Abstract

Marginalized communities, including lesbian and gay populations, may experience unique experiences and expectations associated with growing older. The present study aimed to provide a preliminary model of how lesbian and gay adults view their own aging and identify psychosocial risk and resilience factors specific to this community. Data are from a pilot study of social perceptions of aging in lesbian and gay middle-aged and older adults, in which 45 gay and 46 lesbian adults participants (ages = 55-87, 70% white) completed questionnaires on their health, perceptions of aging, community engagement and identity congruence, and stressful experiences. Self-perceptions of aging were measured with an 8 item scale ranging from 1 to 6, where higher scores indicate greater agreement with positive perceptions of aging (e.g., “So far, I am satisfied with the way that I am aging.”). In a regression model accounting for self-rated health, subjective socioeconomic status, and race/ethnicity, lesbian women reported better self-perceptions of aging than gay men (b = 0.50, SE = 0.16, p <.01). Further, greater outness was associated with better self-perceptions of aging (b = 0.04, SE = 0.01, p <.01), LGBTQ+ community engagement did not relate to self-perceptions of aging (b = -0.10, SE = 0.12, p =.40), and more stressful experiences in daily life were associated with worse self-perceptions of aging (b = -0.15, SE = 0.05, p <.01). Results will be discussed in the context of a model of risk and resilience for “accelerated social aging” among LGBTQ+ populations.

